# Genome-Wide Association Study for Weight-Related Traits in *Scylla paramamosain* Using Whole-Genome Resequencing

**DOI:** 10.3390/ani15131829

**Published:** 2025-06-20

**Authors:** Lin Chen, Yaodong Zhang, Peitan Jia, Siyi Zhou, Qionghui Qin, Weiren Zhang, Kewei Huang, Xiaopeng Wang, Haihui Ye

**Affiliations:** State Key Laboratory of Mariculture Breeding, Fisheries College of Jimei University, Xiamen 361021, China; 202212951095@jmu.edu.cn (L.C.); 202311710025@jmu.edu.cn (Y.Z.); 202211710041@jmu.edu.cn (P.J.); 202311908010@jmu.edu.cn (S.Z.); 202311710017@jmu.edu.cn (Q.Q.); 13124276204@163.com (W.Z.); 202411908055@jmu.edu.cn (K.H.)

**Keywords:** weight-related traits, *Scylla paramamosain*, whole-genome resequencing, GWAS

## Abstract

Weight traits serve as key economic indicators for assessing growth performance and commercial quality in the mud crab *Scylla paramamosain*, yet the genetic basis of these traits remains poorly characterized. Here, we performed whole-genome resequencing on 323 individuals and conducted genome-wide association studies (GWAS) on five weight-related traits: (1) body-related traits, including body weight (BW), trunk weight (TruW), and weight excluding chelae (WEC); (2) appendage-related traits, containing appendage weight (AppW) and cheliped weight (CheW). Significantly associated SNPs were primarily enriched on chromosomes 15, 22, 25, and 36. For body-related traits, we identified 45 shared candidate SNPs and 175 common candidate genes; appendage-related traits revealed 71 shared candidate SNPs, and 229 common genes were identified; and across all five traits, there were 9 shared candidate SNPs and 49 common genes. Gene Ontology (GO) and Kyoto Encyclopedia of Genes and Genomes (KEGG) analyses indicated that shared functional terms/pathways among the five traits were mainly related to metabolism, development, and immunity. Body-related traits exhibited more unique GO terms and KEGG pathways associated with metabolism and immunity, whereas appendage-related traits showed some unique GO terms and KEGG pathways involved in development and morphogenesis. Among the candidate genes, we identified multiple genes associated with growth and development, metabolism, and immune responses. For example, the *CCHa1R* gene, common to carapace-related traits, is linked to feeding; the *DCX-EMA* gene, which is common to appendage-related traits, is connected to movement, and the *MSTO1* gene is pertinent to muscle development. Among the candidate genes shared by all five traits, there are a series of genes concerning growth and development (such as *NVD*, *CYP307A1*, *FGF1*, *NF2*, *ANKRD52*) and immune responses (*RGS10*). These findings advance our understanding of the genetic architecture underlying decapod crustacean growth and provide valuable insights for optimizing sustainable breeding strategies in *S. paramamosain*.

## Simple Summary

In this study, we investigated the genetic basis of weight-related traits in the mud crab *Scylla paramamosain* through whole-genome resequencing of 323 individuals and subsequent genome-wide association studies (GWAS). We analyzed five weight traits: body weight, trunk weight, weight excluding chelae, cheliped weight, and appendage weight. Our results revealed that significant SNPs were primarily concentrated on chromosomes 15, 22, 25, and 36. We identified shared candidate genes for both body-related and appendage-related traits, as well as across all five traits. These candidate genes are clustered in functional categories related to growth, development, metabolism, and immunity. Key genes included *CCHa1R* (related to feeding), *DCX-EMA* (linked to movement), *MSTO1*, *NVD*, *CYP307A1*, *FGF1*, *NF2*, *ANKRD52* (relevant to growth and development), and *RGS10* (associated with immune responses). These findings improve our comprehension of mud crab growth and provide insights for sustainable breeding programs.

## 1. Introduction

*Scylla paramamosain*, commonly known as the mud crab, belongs to the genus *Scylla*, family Portunidae, order Decapoda, and class Crustacea. This species is characterized by a robust carapace (exoskeleton) with serrated edges along the anterior margin. *Scylla paramamosain* typically inhabits intertidal zones with muddy or sandy substrates, where it feeds primarily on slow-moving or benthic organisms such as mollusks, small crabs, and worms [1]. It is widely distributed across the Indo-West Pacific region [2] and is renowned for its large size, flavorful meat, and rich nutritional value [3]. This species plays a significant role in the marine economy of China and Southeast Asia, and holds an important position in aquaculture [4]. In 2023, China’s mud crab aquaculture production reached approximately 160,000 tons [5], constituting over half of the global total production reported by the Food and Agriculture Organization of the United Nations (FAO) [3]. Despite this, the majority of mud crab seeds for aquaculture are still sourced from wild capture, which has led to overfishing concerns in many areas [6]. Furthermore, the lack of high-quality mud crab breeds complicates efforts to meet the increasing demand from the aquaculture sector. Consequently, it is imperative to strengthen genetic breeding efforts, investigate the genetic mechanisms underlying economically relevant traits, and apply this knowledge to breeding practices in *S. paramamosain*. Such initiatives are crucial for advancing the mud crab industry and ensuring the sustainable management of marine biological resources.

Growth traits are critical economic traits for aquatic species, as they directly affect aquaculture efficiency and market value [7,8]. Among these traits, body weight serves as a key representative of growth performance, making it a target for selection in breeding programs aimed at fast-growing and large body sizes. The genetic basis of body weight, being a complex quantitative trait influenced by multiple genes, presents challenges for genetic analysis. However, the rapid advancements in sequencing technologies now enable the detailed investigation of genetic variants and genes associated with weight traits at the whole-genome level in aquatic animals. Among the various phenotypic genetic analysis methods, conducting a genome-wide association study (GWAS) has proven to be a powerful, high-resolution tool for identifying genetic variants linked to complex weight-related traits in fish species, including Atlantic salmon (*Salmo salar*) [9], rainbow trout (*Oncorhynchus mykiss*) [10], olive flounder (*Paralichthys olivaceus*) [11], European sea bass (*Dicentrarchus labrax*) [12], spotted sea bass (*Lateolabrax maculatus*) [13], and brown-spotted grouper (*Epinephelus tauvina*) [14]. In contrast, GWAS research in crustaceans began more recently [15], but has progressed rapidly. For instance, GWAS in the Pacific white shrimp (*Litopenaeus vannamei*) have detected SNPs related to growth [16] and sex determination [17], while research in the oriental river prawn (*Macrobrachium nipponense*) has uncovered candidate genes linked to growth traits [18]. Additionally, GWAS in the Chinese mitten crab (*Eriocheir sinensis*) has revealed SNPs and genes associated with hypoxia tolerance [19], and a study in freshwater crustaceans has explored the *Alpha*, *alpha-trehalose-phosphate synthase (TPS)* gene related to salinity tolerance [20]. Despite these advancements, genetic analysis of phenotypic traits in mud crabs has lagged, primarily due to the long-standing absence of a high-quality reference genome. However, the recent publication of high-quality reference genomes for *S. paramamosain* has provided a solid foundation for analyzing the genetic variations in traits from the whole-genome variation level [1,21]. For example, Zhang et al. released the highest-quality version of the *S. paramamosain* reference genome to date, and identifying a sex-linked region on chromosome 6 through resequencing data [21]. Similarly, Ye et al. performed a genome-wide association study on 100 individuals using 40K SNP microarray data from *S. paramamosain* and identified SNPs and genes related to traits such as body weight, body length, and body height [22]. Despite these efforts, to date, no GWASs have been conducted on weight-related traits in *S. paramamosain* using whole-genome resequencing data.

In this study, we performed whole-genome resequencing on 323 *S. paramamosain* individuals and subdivided weight traits into five specific traits: body weight (BW), trunk weight (TruW), weight excluding chelae (WEC), cheliped weight (CW), and appendage weight (AppW). We utilized GWAS technology to deeply mine genetic loci and candidate genes related to these traits. This study not only provides a new perspective for analyzing the genetic mechanisms of growth traits in crustaceans, but also lays a solid theoretical foundation for the precise breeding of high-yielding new breeds of *S. paramamosain*.

## 2. Materials and Methods

### 2.1. Sample and Phenotype Collection

In October 2023, 323 *S. paramamosain* individuals, including 178 males (55.11%) and 145 females (44.89%), were collected from Fuzhou, Fujian Province, China (Table S1). To minimize environmental variance, all juvenile crabs were cultivated in a single pond system under standardized protocols, maintaining identical initial size distributions and rearing conditions (180 ± 7 days growth period). Harvested crabs were transported to the laboratory at Jimei University within 12 h for dissection to obtain muscle tissue samples. Tissue samples were immediately frozen in liquid nitrogen and stored at −80 °C.

**Figure 1:**
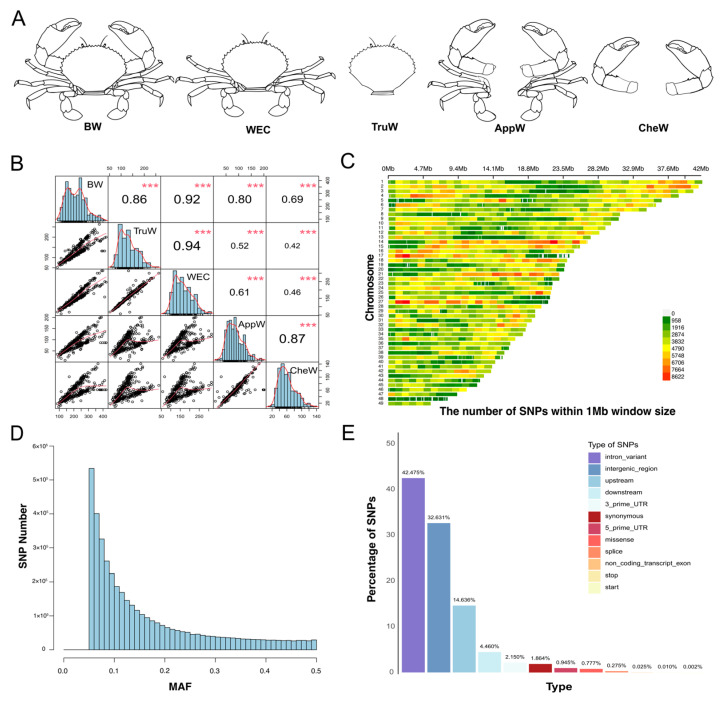
Statistics of phenotypic and sequencing data. (**A**) Five weight-related traits of *S. paramamosain*. The abbreviations BW, WEC, TruW, AppW, and CheW correspond to body weight, weight excluding chelae, trunk weight, appendage weight, and cheliped weight, respectively. (**B**) Correlation among the five traits. *** indicates a *p*-value less than 0.001. (**C**) Density plot of SNP distribution across chromosomes based on sequencing data, generated using the R package CMplot (v4.5.1) [23]. (**D**) Distribution plot of the minor allele frequency (MAF) counts for SNPs. (**E**) Classification information of SNPs after annotation.

Weight-related traits were classified into five categories based on the body configuration of *S. paramamosain* (Figure 1A): (1) body weight (BW), the weight of the entire body; (2) weight excluding chelae (WEC), the body weight after removing the two large chelae; (3) trunk weight (TruW), the body weight minus the appendage attached to the cephalothorax; (4) cheliped weight (CheW), the combined weight of the two large chelae; and (5) appendage weight (AppW), the weight of all ten legs. The Wilcoxon rank-sum test was employed to assess the statistical significance of distributional differences in five traits across sexes, with results visualized using the R programming language.

### 2.2. DNA Extraction, Sequencing, and Variant Calling

Genomic DNA was isolated using TaKaRa DNA extraction kits (Takara Biotechnology Co., Kusatsu, Japan) following the manufacturer’s protocol. The concentration and quality of the extracted DNA were assessed using a NanoDrop 2000 Spectrophotometer (Thermo Fisher Scientific, Waltham, MA, USA). DNA libraries with an average insert size of 350 bp were prepared following Illumina standard protocols and sequenced on an Illumina HiSeq X plus platform by a commercial service provider (Novogene, Beijing, China) to generate 150 bp paired-end reads.

Raw sequencing reads in FASTQ format were processed using *fastp* (v0.23.2) [24]. In this step, adapter sequences, reads containing ploy-N regions, and low-quality reads were removed to obtain high-quality clean reads. Additionally, Q20, Q30, GC content, and sequence duplication levels were calculated to assess data quality. The filtered reads were mapped to the reference genome [21] using *BWA* (v0.7.12) [25]. The alignment results were sorted, and duplicate reads were marked using *Samtools* (v1.9) [26]. Variant calling, including single-nucleotide polymorphisms (SNPs) and insertions/deletions (InDels), was performed using the HaplotypeCaller module in *GATK* (v3.8) [27] with the following filtering criteria: QD < 2.0 || MQ < 40.0 || FS > 60.0 || QUAL < −12.5 || ReadPosRankSum < −8.0-clusterSize 2-clusterWindowSize 5. SNPs annotation was performed on the basis of the reference genome using *snpEff* (v3.6c) [28]. Variants were categorized into intergenic regions, upstream or downstream regions, exons or introns. SNPs located in coding regions were further classified as synonymous or nonsynonymous.

For quality control, *PLINK* (v1.9) [29] was employed to trim the data with minor allele frequency (MAF) < 0.05, call rates < 90%, and Hardy–Weinberg equilibrium (HWE) *p*-values < 0.000001. After filtering, a final set of 4,042,299 high-confidence SNPs from 323 individuals was retained for subsequent analysis.

### 2.3. Phenotypic Heritability and Correlations

Heritability ($h^{2}$) of traits was defined as the ratio of the additive genetic variance to phenotypic variance. The SNP-based heritability (*h*^2^) was calculated using *HIBLUP* software (v1.5.3) [30] as follows:

$$h^{2}=\frac{\sigma_{a}^{2}}{\sigma_{a}^{2}+\sigma_{e}^{2}}$$

where $\sigma_{a}^{2}$ represents the additive genetic variance and $\sigma_{e}^{2}$ represents the residual variance. Furthermore, Pearson correlation coefficients were computed for pairs of traits to further investigate the correlations among the phenotypic characteristics themselves. Trait correlations were visualized using the “chart.Correlation” function in the *PerformanceAnalytics* (v2.0.4) [31] package of the R programming language.

### 2.4. Population Structure Analysis

Principal component analysis (PCA) was conducted using *PLINK* (v1.9) [29], and the results were visualized through *ggplot2* (v3.5.1) [32] package in R. A genomic relationship matrix (GRM) was computed utilizing *GCTA* (v1.94.3) [33], and a heatmap was generated with the *pheatmap* (v1.0.12) [34] package in R.

### 2.5. Genome-Wide Association Study

Genome-wide association studies (GWAS) were conducted to examine associations between genome-wide SNPs and individual weight-related traits using *GEMMA* (v0.98.5) [15]. The univariate linear mixed model (LMM) was applied as follows:

$$y=W\alpha +x\beta +u+\varepsilon ;\;u\sim {\mathrm{MVN}}_{n}(0,\;\lambda \tau^{-1}K),\;\varepsilon \sim {\mathrm{MVN}}_{n}(0,\tau^{-1}l_{n})$$

where *y* is the phenotype vector; *W* is the fixed effect matrix, including the top six eigenvectors of PCA, sex, and date of sampling; α is a c-vector of the corresponding coefficients including the intercept; *x* is the vector of SNP genotype; β indicates the effect size of the marker; *u* is a random effect; ε represents residuals; *λ* is the ratio between the two variance components; τ^−1^ is the residual variance; *K* is the standardized correlation matrix estimated by *GEMMA* (v0.98.5) [15]; *l_n_* is the unit matrix; MVN_n_ denotes the n-dimensional multivariate normal distribution; and *n* is the number of animals.

Considering the stringency of the Bonferroni correction threshold (*p* = 1.24 × 10^−8^; 0.05/4,042,299), this study adopted a more lenient threshold of *p* = 1.0 × 10^−5^, which commonly recommended for GWAS discoveries [35,36,37]. The phenotypic variance explained (PVE) by each significant locus was estimated using the methodology of previously study [38]. To systematically evaluate the statistical power of our GWAS, we conducted power analysis using G*Power software (version 3.1.9.7) [39]. A two-tailed *t*-test was employed with the “Linear multiple regression: Fixed model, single regression coefficient” module, and PVE was used as the effect size metric [40].

### 2.6. Gene Annotation and Functional Enrichment Analysis

According to previous research [13,41,42], candidate genes were identified within 100 kb upstream and downstream of significant SNPs using the R package *GALLO* (v1.5) [43]. Protein sequences from the reference genome were annotated using *EggNOG-mapper* (v2.1.12) [44], and a custom database was built with the R package *AnnotationForge* (v1.44.0) [45]. Gene Ontology (GO) and Kyoto Encyclopedia of Genes and Genomes (KEGG) enrichment analyses were performed using the R package *clusterProfiler* (v4.10.1) [46]. The enriched terms with the criteria of *p* < 0.01 were selected to further explore the genes involved in pathways and biological processes.

## 3. Results

### 3.1. Measurement of Phenotypic and Genomic Data

Phenotypic data for 323 individuals showed a normal distribution (Figure 1B and Figure S1). Specifically, the mean ± standard deviation (SD) for BW was 218.74 ± 74.37 g, TruW was 132.07 ± 45.35 g, WEC was 157.66 ± 53.28 g, AppW was 86.67 ± 38.59 g, and CheW was 61.08 ± 30.90 g (Table 1), respectively. Pearson’s correlation analysis revealed correlation coefficients greater than 0.86 among BW, TruW and WEC traits, and 0.87 between CheW and AppW (Figure 1B). Sex-stratified correlation analysis confirmed consistency with the combined-sex results: BW, TruW, and WEC showed strong correlations, while CheW and AppW also exhibited highly significant correlations (Figure S1). Based on their body parts and correlations, the traits were classified into two groups: (1) the BTW group (body-related traits, comprising BW, TruW, and WEC), and (2) the AC group (appendage-related traits, containing CheW and AppW). The results of the Wilcoxon rank-sum test revealed significant sexual dimorphism in four out of five traits (all *p* < 0.01), except for BW (*p* = 0.4). Specifically, females showed higher TruW (*p* = 5.5 × 10^−5^) and WEC (*p* = 0.0017), but lower AppW (*p* = 1.9 × 10^−14^) and CheW (*p* < 2 × 10^−16^) than males (Table 1 and Figure S2). These findings necessitate sex adjustment in subsequent GWAS analyses.

The dataset utilized for this analysis comprises 3.5 TB of clean data, achieving a Q30 score of 91.11%. The effective rate was 96.86%, average GC content was 42.39%, and average coverage depth of 9× (Table S1). *GATK* (v3.8) identified a total of 55,230,846 SNPs, of which 5,572,631 were shared across all individuals with complete data (no missing calls), and 49,718,299 SNPs remained after hard filtering. The analysis identified 41,766,627 SNPs with MAF < 0.05, representing 84% of the SNPs subjected to hard-filtering criteria. After quality control by *PLINK* (v1.9) [29] and phase and imputation by *Beagle* (v5.5) [47], a total of 4,042,299 SNPs were obtained, which were evenly distributed across all chromosomes without large gaps except chromosome 48 (Figure 1C), and the average distance between SNPs was 300 bp. These SNPs were used in subsequent analyses.

We constructed the site frequency spectrum of the high-quality SNPs (Figure 1D). This spectrum demonstrates an L-shaped distribution (Figure 1D), indicating that as the MAF increases, the corresponding number of sites decreases. After conducting annotation analysis on these high-quality SNPs across all 323 individuals, we identified that the four most prevalent types of SNPs are intron (42.48%), followed by intergenic (32.63%), upstream (14.64%), and downstream (4.46%).

### 3.2. Population Structure

**Figure 2:**
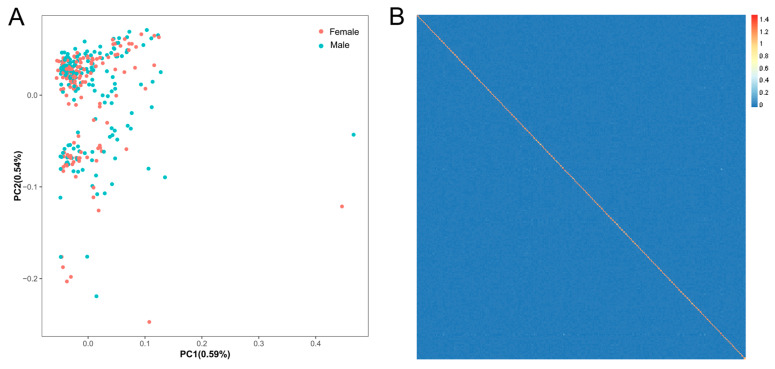
Population genetic structure analysis. (**A**) Principal component analysis; red and blue dots represent female and male individuals, respectively; (**B**) heatmap of the genetic relationship matrix for the tested population.

PCA results indicated no significant genetic disparities among individuals, with PC1 and PC2 explaining 0.59% and 0.54% of the variance (Figure 2A), respectively. Consistent with the PCA results, the heatmap of the genetic relationship matrix revealed small genetic differences among individuals (Figure 2B), suggesting that there is no significant population stratification in the population.

### 3.3. GWAS Results

In this study, 4,042,299 SNPs from 323 *S. paramamosain* samples were retained for GWAS analysis. Given the conservative nature of the genome-wide significance threshold (1.24 × 10^−8^, calculated as 0.05 divided by 4,042,299) derived from strict application of Bonferroni correction in GWAS results, this study employed a relatively lenient significance threshold of *p* < 10^−5^, which has been widely accepted as reliable in numerous studies [35,37,48].

**Figure 3:**
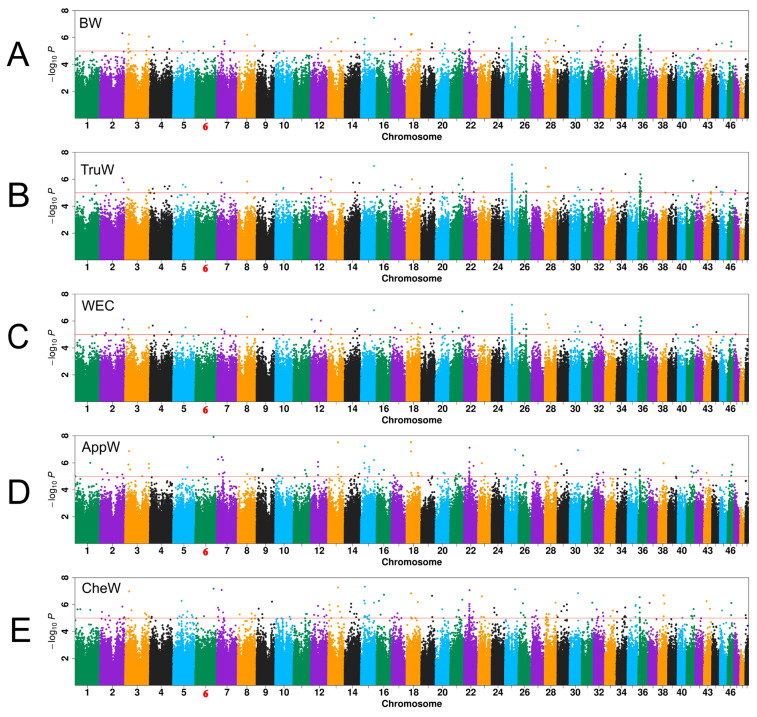
Manhattan plots from genome-wide association studies. (**A**) Body weight (BW); (**B**) trunk weight (TruW); (**C**) weight excluding chelae (WEC); (**D**) appendage weight (AppW); (**E**) cheliped weight (CheW). Red dashed lines indicate the significance threshold (*p* = 10^−5^). Chromosome 6 (highlighted in red) corresponds to the sex chromosome as previously reported [21].

The quantile–quantile (Q-Q) plot was employed to compare the distribution of observed log_10_(*p*) values for whole-genome SNPs to the theoretical distribution of expected values. The genomic inflation factor (λ) of these traits ranged from 0.993 to 1.011 (Figure S1), indicating that the effects of population stratification in the GWAS analytical model were reasonably corrected. For the traits of BW, TruW, WEC, AppW, and CheW, 101, 104, 99, 109, and 167 significant SNPs were identified across 30, 34, 34, 38, and 44 chromosomes, respectively (Table S2, Figure 3). Chromosomes 25 and 36 emerged as hotspots for SNPs related to BW, TruW, and WEC, while chromosomes 15 and 22 were notable for AppW and CheW. The PVE for significant association loci (*p* < 10^−5^) for five weight-related traits ranged from 6.24% to 10.97%, with a mean value of 7.05% (Table S2). Statistical power analysis revealed that detecting variants with an effect size equivalent to the mean PVE (7.05%) would require approximately 400 samples to achieve 80% power (power = 0.8). Under the actual sample size (n = 323), the detection power for variants with PVE = 7.05% is estimated at 60%. Notably, variants with larger effect sizes (e.g., PVE > 8.8%) still achieve over 80% power under the current sample size, demonstrating reliable detection capability for genetic variants with moderate-to-large effects.

**Figure 4:**
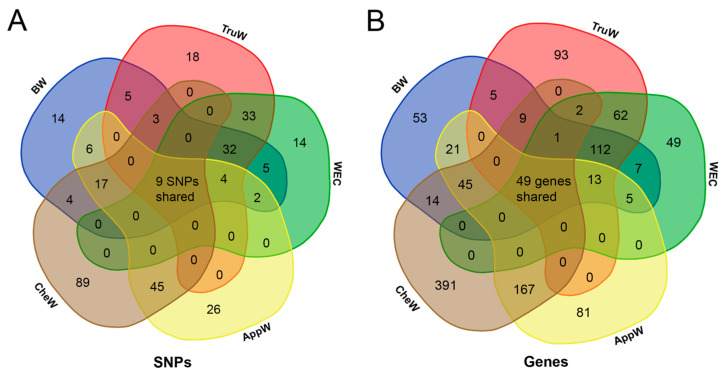
Venn diagram analysis of GWAS results. (**A**) Shared significant SNP loci across five traits. Numerical values within intersection areas denote the quantity of shared SNPs, with the central overlap indicating nine SNPs common to all five traits. (**B**) Shared candidate genes across five traits. Numerical values within intersection areas denote the quantity of shared genes, with the central overlap indicating 49 candidate genes common to all five traits. The full names corresponding to the five phenotypic abbreviations can be found in Table 1.

Following gene annotation, 334, 346, 300, 381, and 678 genes were annotated for BW, TruW, WEC, AppW, and CheW traits (Table S3), respectively. In the BTW group, 93 significant SNPs were detected by at least two of three traits (Figure 4A), with 45 significant SNPs and 175 candidate genes shared by all three traits (Figure 4B). Similarly, in the AC group, 71 significant SNPs were common to both traits (Figure 4A), annotating 229 shared candidate genes (Figure 4B). Remarkably, nine significant SNPs were shared by all five traits (Figure 4A), primarily located on chromosomes 2, 3, 15, 25, 26, 30, and 36, and these SNPs were annotated to 47 shared genes (Figure 4B, Table 2). In addition, two candidate genes were identified as being shared among the five phenotypic candidate regions, albeit without sharing any SNPs (Table 2). Among the nine candidate SNPs, seven are intergenic variants, and two are located within introns of the uncharacterized genes *LOC135113241* and *ANKRD52*, respectively. LD analysis revealed that the three SNPs on chromosome 3 reside within a 143 bp LD block (r^2^ > 0.8, Figure S5), whereas the other six inter-chromosomal SNPs exhibit low LD (r^2^ < 0.1), indicating that these signals may reflect independent genetic effects (Table S4).

### 3.4. Functional Annotation

GO function analysis and KEGG pathway analysis are pivotal in investigating gene function and elucidating biological processes. In this study, we conducted GO and KEGG analyses on all candidate genes associated with the five traits to explore their functional roles. The candidate genes were mainly enriched in terms and pathways related to metabolism, growth, and immunity.

**Figure 5:**
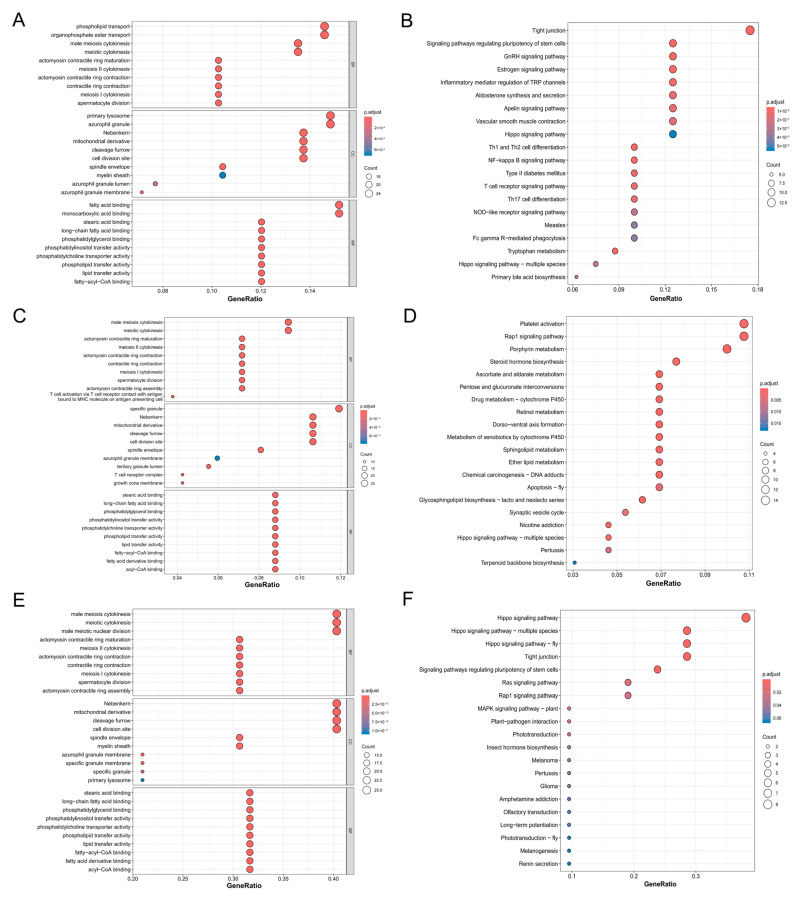
Functional enrichment analysis of candidate genes. The top 10 significant GO terms for biological process (BP), cellular component (CC), and molecular and function (MF) categories are presented for common candidate genes in the (**A**) BTW group, (**B**) AC group, and (**C**) the genes common to both BTW and AC groups. Additionally, the top 20 significant KEGG pathways are displayed for shared candidate genes in the (**D**) BTW group, (**E**) AC group, and (**F**) the genes common to both BTW and AC groups.

For the traits of the BTW group, a total of 757 GO terms were detected, including 616 biological process (BP) terms, 53 cellular component (CC) terms, and 88 molecular function (MF) terms (Figure 5A, Table S5). For the AC group, 429 GO terms were detected, encompassing 340 BP terms, 45 CC terms, and 40 MF terms (Figure 5C, Table S5). There were 155 GO terms shared between the two groups (Figure 5E, Table S5), which primarily involved biological processes such as actomyosin development (e.g., GO:0031566 actomyosin contractile ring maturation; GO:0000916 actomyosin contractile ring contraction), germ cell division (e.g., GO:0007112 male meiosis cytokinesis; GO:0007111 meiosis II cytokinesis), lipid transport (e.g., GO:0015914 phospholipid transport; GO:0110112 regulation of lipid transporter activity), skeletal development (e.g., GO:0048706 embryonic skeletal system development; GO:0048705 skeletal system morphogenesis), organogenesis (e.g., GO:0048703 embryonic viscerocranium morphogenesis; GO:0048645 animal organ formation), immunity (e.g., GO:0035722 interleukin-12-mediated signaling pathway; GO:0032740 positive regulation of interleukin-17 production), optesthesia (GO:0046669 regulation of compound eye retinal cell-programmed cell death; GO:0061074 regulation of neural retina development), and olfaction (GO:0021553 olfactory nerve development). In terms of cellular components, the enriched terms mainly related to cellular energy metabolism and differentiation (e.g., GO:0016006 Nebenkern; GO:0005766 primary lysosome), immune response (e.g., GO:0042582 azurophil granule; GO:0035577 azurophil granule membrane; and GO:0042581 specific granule), etc. For molecular functions, they primarily concerned lipid and fatty acid binding (e.g., GO:0070540 stearic acid binding; GO:0005504 fatty acid binding) and phospholipid transport (e.g., GO:0005548 phospholipid transporter activity; GO:0120013 lipid transfer activity), etc.

A comparison of the GO analysis results between the BTW and AC groups revealed that the GO terms of the BTW group contained more terms related to metabolism and immunity in biological processes. In contrast, the AC group was enriched with numerous terms relevant to development and morphogenesis, particularly terms related to limb development, such as GO:0035292, specification of segmental identity, and trunk. In terms of cellular components, the BTW group was enriched with more terms related to immune response (e.g., GO:0001772 immunological synapse; GO:0031091 platelet alpha granule) and membrane-associated components (e.g., GO:0033116 endoplasmic reticulum–Golgi intermediate compartment membrane; GO:0005798 Golgi-associated vesicle). For molecular functions, the BTW group was enriched with more GO terms related to enzymatic activity (e.g., GO:0003997 acyl-CoA oxidase activity; GO:0016505 peptidase activator activity involved in apoptotic process), as well as receptor and ligand binding (e.g., GO:0043560 insulin receptor substrate binding; GO:0051379 epinephrine binding).

For the candidate genes shared by the BTW and AC groups, 38 and 30 KEGG pathways were detected (Figure 5B,D, Table S6), respectively, which were mainly related to immunity, metabolism, and development. The shared KEGG pathways between the two groups primarily involved metabolism and growth (e.g., ko00040: pentose and glucuronate interconversions; ko04390: Hippo signaling pathway) (Figure 5F). The BTW group-specific KEGG pathways mainly included immune response (ko04658: Th1 and Th2 cell differentiation; ko04660: T cell receptor signaling pathway), sex hormone signaling (ko04912: GnRH signaling pathway; ko04915: estrogen signaling pathway), fatty acid degradation and metabolism (ko00071: fatty acid degradation; ko01212: fatty acid metabolism), etc. In contrast, the AC group-specific KEGG pathways primarily comprised vitamin metabolism (ko00053: ascorbate and aldarate metabolism; ko00830: retinol metabolism; and ko00750: vitamin B6 metabolism).

### 3.5. Key Candidate Area

**Figure 6:**
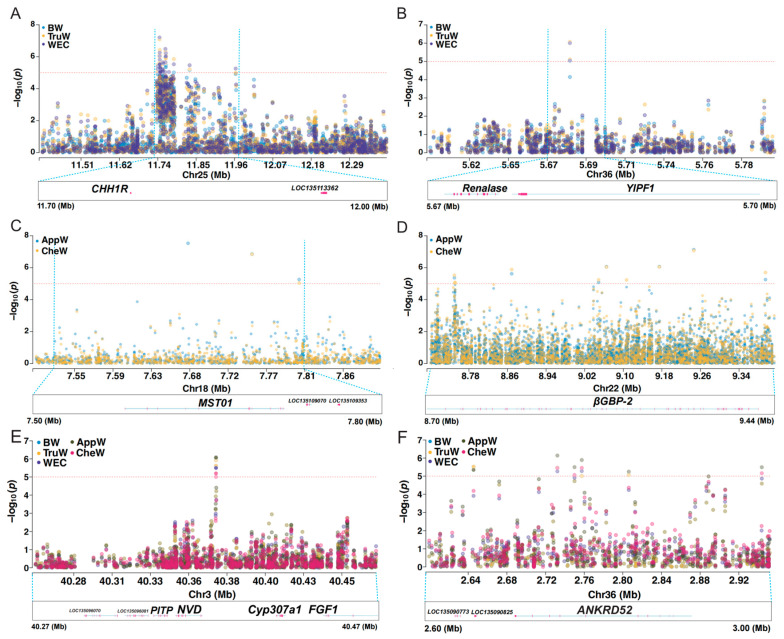
Genome-wide association analyses reveal pleiotropic loci for growth-related traits in mud crab. Manhattan plots highlighting significant genomic regions associated with phenotypic traits: (**A**) chromosome 25 (11.40–12.40 Mb), shared association with BW, TruW, and WEC; (**B**) chromosome 36 (5.60–5.80 Mb), shared association with BW, TruW, and WEC; (**C**) chromosome 18 (7.48–7.90 Mb), shared association with AppW and CheW; (**D**) chromosome 22 (8.70–9.44 Mb), shared association with AppW and CheW; (**E**) chromosome 3 (40.26–40.47 Mb), pleiotropic region influencing all five traits; (**F**) chromosome 36 (2.60–3.00 Mb), pleiotropic region influencing all five traits. Red dashed line indicates the significance threshold (*p* = 1 × 10^−5^). The full names corresponding to the five phenotypic abbreviations can be found in Table 1. Blue dashed lines demarcate the physical position interval in the Manhattan plot (**upper panel**), which directly corresponds to the physical position range displayed in the **lower panel**.

In this section, we focused on the common regions among three traits in the BTW group, the candidate regions shared by the two traits in the AC group, and the candidate regions that are ubiquitous across all five traits (Figure 6). Within the common regions of the BTW group, 11 candidate SNPs were concentrated in the chr25: 11,640,576–11,928,077 region, which harbors the appetite-related gene *CCHa1R* (neuropeptide CCHamide-1 receptor) (Figure 6A). Additionally, one candidate SNP was mapped to the 5,581,593–5,781,593 region of chromosome 36, which encompasses the gene *YIPF1* (Yip1 domain-containing) (Figure 6B).

In the shared regions of the AC group, we identified four significant SNPs within the 5,490,605–6,763,411 region of chromosome 15, which contains the gene *DCX-EMAP* (Doublecortin domain-containing echinoderm–microtubule-associated protein). Furthermore, two significant SNPs were found in the 7,651,246–7,905,855 region of chromosome 18, which includes the *MST01* (Misato mitochondrial distribution and morphology regulator) gene (Figure 6C). Additionally, eight significant SNPs were concentrated in the 8,575,029–9,499,114 region of chromosome 22, which harbors the immune-related gene *BGBP2* (beta-glucan-binding protein 2) (Figure 6D).

In the common regions of BTW and AC groups, three SNPs were concentrated in the 40,271,752–40,471,757 region of chromosome 3, which includes the genes *PITP* (phosphatidylinositol transfer protein), *NVD* (cholesterol 7-desaturase nvd), *CYP307A1* (cytochrome P450 307A1), and *FGF1* (fibroblast growth factor 1) (Figure 6E). One SNP was located in the 2,632,612–2,832,612 region of chromosome 36, containing the gene *ANKRD52* (serine/threonine protein phosphatase 6 regulatory ankyrin repeat subunit C) (Figure 6F). Additionally, there was one candidate SNP in each of the chr2: 37,715,876–37,815,876, chr15: 22,304,281–22,504,281, chr25: 17,254,201–17,454,201, chr26: 8,689,084–8,889,084, and chr30: 14,347,661–14,547,661 regions (Table 2). These QTL regions encompass the genes *DLX* (homeotic protein Distal-less), *ERGIC1* (endoplasmic reticulum–Golgi intermediate compartment protein 1), *NaCh* (sodium channel protein), *HPGD* (5-hydroxyprostaglandin dehydrogenase [NAD(+)]), *RGS10* (regulator of G protein signaling 10), and *ZCRB1* (zinc finger CCHC-type and RNA-binding motif-containing protein 1).

## 4. Discussion

Weight traits are among the most economically significant attributes in aquaculture species, making them a consistent focus of genetic research. In crabs, these traits are not limited to total body weight but are also significantly influenced by the weights of their carapace, cheliped, and appendages—key contributors to the value of crab meat. Hence, identifying genetic loci and genes associated with these traits is essential for advancing selective breeding programs and improving economic traits in crabs. Although the draft genome and transcriptome resources for the mud crab *S. paramamosain* have been established [1,21], genome-wide association studies (GWAS) on its economic traits remain limited. Most previous studies have relied on candidate gene screening or linkage analysis [49,50,51,52], which offer a narrow perspective on the complex multigenic regulation of traits. Advances in whole-genome resequencing (WGR) technology now enable high-precision GWAS by generating dense genome-wide SNP markers, particularly valuable for species with complex genetic backgrounds. In this study, we used WGR data (approximately 9× coverage) from 323 *S. paramamosain* individuals and conducted GWAS to identify genetic variation loci and genes associated with five weight-related traits, providing insights into the genetic mechanisms underlying these traits.

Population genetic structure analyses revealed small genetic differences among individuals, which were supported by the genetic distance matrix and PCA. To correct for potential population stratification, PCA was included as a covariate in the GWAS model, which was validated by λ values indicating effective correction. Pearson’s correlation analysis revealed strong genetic correlations among BW, WEC, and TruW (correlation coefficients > 0.86), as well as between App and CheW (correlation coefficient = 0.87). Based on these correlations, we categorized the traits into two groups, BTW and AC, to explore their shared and unique genetic bases. Four traits (excluding BW) exhibited significant sex-specific variation (*p* < 0.01). However, given the current sample size constraints (178 males vs. 145 females), sex-stratified GWAS would lack sufficient statistical power to reliably detect genetic associations. We therefore systematically incorporated sex as a fixed-effect covariate within our LMM framework to mitigate confounding effects arising from sex-related biases. This methodological strategy has been established as a standard practice in studies of sexually dimorphic traits across diverse species, including humans [53,54,55], livestock [48,56,57,58], and aquatic species [8,9,22,59]. Future investigations will expand the cohort to approximately 1000 individuals per sex to enable systematic dissection of sex-specific genetic regulatory mechanisms underlying phenotypic sexual dimorphism. A significance threshold of *p* < 10^−5^ was selected for GWAS, due to the excessive stringency of the Bonferroni correction. This threshold allowed us to identify SNPs and genes with both statistical significance and potential breeding value.

GO analysis revealed both mutual and special terms in the BTW and AC groups. Shared GO terms included those related to actin and skeletal development, highlighting their role in muscle growth and weight improvement. Immune-related GO terms were enriched in both groups, suggesting that immune processes play a critical role in regulating body weight and appendage weight. Metabolic pathways, such as fatty acid metabolism and energy balance, were also shared, reflecting the metabolic regulation of growth and appendage specialization. Interestingly, vision- and olfaction-related GO terms were identified, hinting at a connection between feeding behavior and weight traits. Distinct pathways were observed between the two groups, aligning with their phenotypic differences. The BTW group showed enrichment in metabolism and immunity-related GO terms, reflecting the heightened genetic regulation necessary for the growth and development of the main body. Conversely, the AC group exhibited enrichment in morphogenesis, organ development, and behavior-related terms, consistent with the functional and morphological roles of appendages. For instance, GO terms related to sex organ development in the AC group corroborate the sexual dimorphism observed in crab appendages, such as male copulatory organs and female reproductive structures.

KEGG analysis further emphasized shared pathways, including the Hippo signaling pathway, which is a crucial regulator of organ size, tissue growth, and regeneration [60,61]. Previous studies have linked the Hippo pathway to size regulation in *Drosophila* eyes [62], wings [63], and mouse liver [64], underscoring its conserved role across species. Comparative genomic analysis of the giant isopod (*Bathynomus jamesi*) revealed significant expansions in growth-related pathways including the Hippo signaling pathway, which may underpin its enormous body size [65]. The presence of some expanded gene families enriched in the Hippo signaling pathway in the genome of the swimming crab (*Portunus trituberculatus*) may be related to salinity adaption and immune stress in this species [66]. Additionally, the phototransduction pathway, involved in vision, was also enriched in both groups, aligning with its importance in feeding and growth. Notably, the enrichment of the phototransduction pathway has also been observed in a previous transcriptomic differential analysis between fast-growing and slow-growing groups of the Pacific white shrimp (*P. vannamei*) [67]. Meanwhile, comparative analysis revealed distinct KEGG pathway profiles between two groups. The BTW group exhibited unique enrichment in metabolic and immune-related pathways (e.g., lipid metabolism, lysosome activity), potentially reflecting their critical roles in systemic energy regulation and body development. In contrast, the AC group demonstrated specialized vitamin metabolism pathways, particularly involving vitamins A–, C–, and B6–micronutrients with established roles in chitin remodeling and skeletal homeostasis, as supported by recent reviews on vitamin–bone interactions [68,69]. These differences suggest that during the developmental process of different body parts in mud crabs, distinct biological pathways and mechanisms may be required to regulate their growth and function.

Comparative analyses of candidate regions revealed significant enrichment of SNP in specific regions: chr25 and chr36 for the BTW group and chr22 and chr36 for the AC group. These shared chromosomal regions may contain pleiotropic loci or genes with shared genetic determinants that simultaneously influence multiple traits within each group, potentially reflecting overlapping biological pathways or regulatory mechanisms. In view of the current limitations in resolving gene functions in the mud crab, this study used a cross-species functional annotation strategy to infer biological functions of candidate genes in mud crab by using gene function data in model organisms and economic aquatic animals. Consistent with expectations, key genes related to growth, development and immunity were annotated in these regions. For instance, the chr25 region (BTW group) harbors the neuropeptide CCHamide-1 receptor gene, which regulates feeding and growth in arthropods [70,71]. For example, suppressing the expression of *CCHa1* or *CCHa1R* through RNA interference technology leads to a significant reduction in feeding and impaired growth and development in the pea aphid *Acyrthosiphon pisum* [71]. On the other hand, the chr15 region common to the AC group contains the *DCX-EMA* gene, which has been shown to be involved in insect locomotion and mechanosensory transduction [72]. The chr18 region contains the *MSTO1* gene, which is relevant to the regulation of mitochondrial distribution and morphology. Mutations in this gene can lead to clinical manifestations of mitochondrial dysfunction, including muscle weakness, short stature, and delayed motor development [73]. Furthermore, the chr22 region comprises the beta-glucan-binding protein 2 gene, which is one of the key categories of pathogen recognition receptors and plays an important role in the immune system of arthropods [74].

Shared candidate genes across the groups are mainly related to growth, development and immunity. For example, the *NVD* gene, essential for ecdysteroid synthesis and growth [75]. Loss of *NVD* function results in arrested molting and growth during *Drosophila* development [75]. Additionally, 7-DHC, a product of *NVD*, is a precursor of vitamin D3, which impacts crustacean growth, molting, and the immune system [76]. The *CYP307A1* gene belongs to the cytochrome P450 (CYP) superfamily of cytochromes, and the CYP family undergoes significant expansion through gene duplication (e.g., tandem duplication) in crustaceans [77,78]. *CYP307A1* is another key gene in ecdysteroid synthesis [79] that is potentially involved in the regulation of insect growth and development [80]. *FGF1*, an important growth factor gene, plays a pivotal role in various biological processes, including accelerating wound healing and promoting tissue regeneration [81,82].

Moreover, other shared regions also contain many functional genes. These include the *Merlin* gene encoding a key upstream regulatory factor in the Hippo signaling pathway. Loss of *Merlin* function leads to Hippo pathway dysregulation, affecting growth and development [83]. The *DLX* gene is crucial for development [84] and can influence insect recognition of specific odors by regulating olfactory-related gene expression [85]. The *HPGD* gene participates in inflammatory response regulation by modulating prostaglandin levels [86]. *RGS10*, a central regulator of the G-protein signaling pathway, affects Th1/Th17-mediated immune responses by modulating STAT1/STAT3 phosphorylation in mammals, suggesting its potential role in innate immune regulation [87]. In crustaceans (e.g., *Litopenaeus vannamei*, *Eriocheir sinensis*, and *Scylla paramamosain*), the Toll, IMD, and JAK/STAT pathways are core immune regulatory networks [88,89,90]. There is cross-synergy among these pathways: JAK/STAT activation by Toll/IMD pathways collectively regulates antimicrobial peptide synthesis [88,91,92]. Although direct experimental evidence of *RGS10* in crustaceans is lacking, its conserved function in STAT signaling (e.g., JAK/STAT-mediated antifungal/antiviral immunity [87,88]) implies that *RGS10* may indirectly influence Toll/IMD signaling via JAK/STAT pathways. For instance, Toll/IMD pathways in decapods rely on NF-κB signaling [89], while JAK/STAT interacts with Toll pathways through interferon-like regulation [88], indicating *RGS10*’s potential regulatory role in such networks. Finally, the *ANKRD52* gene, located in the region of chromosome 36, is associated with height and body mass index (BMI) [93,94]. The candidate genes shared across the five traits are primarily related to growth, development, and immunity. These genes may modulate holistic growth-related networks, thereby influencing weight-related traits in each body part. These findings not only deepen our understanding of modular growth in decapod crustaceans, but also support diverse breeding strategies for *S. paramamosain* by identifying both shared and unique phenotypic genes.

Weight-related traits represent classic complex traits governed by gene–environment interplay [95,96]. Although our study strictly controlled macro-environmental conditions through standardized rearing protocols (uniform larval size, pond systems, and feeding management), residual micro-environmental variations and individual physiological differences may still introduce unmeasured environmental noise into GWAS analyses. For instance, individual feeding behavior variations could affect weight traits through differential nutrient intake efficiency, while micro-fluctuations in temperature might interact with growth-related phenotypes via epigenetic modifications or metabolic pathway regulation. Future studies could adopt precision agriculture technologies, such as deploying real-time environmental monitoring sensors (e.g., water temperature/oxygen loggers) and individual behavioral tracking systems (e.g., RFID feeding monitors), to construct linear mixed models incorporating micro-environmental variables. This would enable the deeper dissection of genotype-by-environment interactions underlying complex traits.

The relatively low SNP-based heritability estimates (h^2^ = 0.20–0.40) likely arise from multiple factors: unaccounted environmental noise, non-additive genetic effects not captured by additive SNP models, limited sample sizes, and phenotypic measurement errors [97,98]. Nevertheless, genome-wide significant loci (*p* < 1 × 10^−5^) show substantially higher PVE (6.24–10.97%; mean 7.05%) than the genome-wide background (mean PVE = 0.3%), indicating their substantial genetic contributions. While power analysis confirms that our sample size is modest, all significant loci exhibit PVE > 6.24%, demonstrating practical genetic significance [99]. Under the current sample size, the statistical power reaches 60% for variants with mean effect size (PVE = 7.05%), which remains practically valuable for candidate gene screening. For loci with greater effect sizes (PVE > 8.8%), the statistical power exceeds 80%, confirming the reliability of our detection capability for significant associations. It is also noteworthy that published GWASs in aquaculture genetics with smaller sample sizes (e.g., n < 400) have similarly gained scientific recognition [8,12,13,22]. This study provides foundational candidate genes for mud crab weight-related traits and critical data for genetic dissection of economically important traits. Future work should incorporate non-additive genetic models, expanded sample sizes, refined environmental covariates, and multi-temporal phenotypic assessments to better disentangle genetic effects from confounding factors and improve detection power.

GWAS-identified SNPs significantly associated with weight-related traits facilitate quantitative trait locus (QTL) mapping, enabling the prioritization of candidate or even causal genes underlying these five traits. Notably, these QTL regions may harbor variants within coding regions (e.g., non-synonymous mutations) or regulatory elements (e.g., promoters, enhancers), which modulate causal gene expression through cis-regulatory interactions, thereby providing direct mechanistic insights into phenotypic determination. Furthermore, in describing the functions of GWAS candidate genes, while cross-species gene function annotations offer plausible mechanistic hypotheses, we emphasize the necessity of direct functional validation in crustacean systems to substantiate these associations and determine causality. Moving forward, targeted experimental validation utilizing integrated multi-omics approaches (e.g., transcriptomics, epigenomics) and molecular biology techniques (e.g., CRISPR/Cas9 gene editing) will be essential to rigorously test the observed gene–phenotype linkages and elucidate their biological significance. Practically, validated SNPs may serve as molecular markers to facilitate early-stage selection in mud crabs, thereby expediting genetic improvement of growth traits through marker-assisted selection (MAS) strategies.

## 5. Conclusions

In this study, we performed genome-wide association analyses for five weight-related traits in the mud crab *S. paramamosain* using whole-genome resequencing data. The results unveiled shared and specific GO terms and KEGG pathways among the five traits, which highlighted their genetic similarities and unique characteristics. Furthermore, we pinpointed common genes associated with growth and immunity across all five traits, along with distinct candidate genes specific to the BTW and AC groups. These promising candidate genes provide a valuable theoretical foundation for the genetic enhancement of weight-related traits in *S. paramamosain*. Further research, including multi-omics studies and functional verification experiments, is imperative to validate and refine the outcomes of this study.

## Figures and Tables

**Table 1:** Summary statistic of weight-related traits in *Scylla paramamosain*.

Trait ^a^	No ^b^	Mean (±SD) ^c^/g	Female: Mean (±SD) ^c^/g	Male: Mean (±SD) ^c^/g	CV (%) ^d^	Female: CV (%) ^d^	Male: CV (%) ^d^	*h* ^2 e^
BW	320	218.74 ± 74.37	213.52 ± 74.31	219.49 ± 66.93	34.00	34.80	30.49	0.32
TruW	317	132.07 ± 45.35	141.93 ± 48.44	119.66 ± 30.44	34.34	34.12	25.43	0.20
WEC	319	157.66 ± 53.28	168.15 ± 58.42	145.79 ± 39.14	33.79	34.74	26.84	0.25
AppW	317	86.67 ± 38.59	69.24 ± 25.97	97.23 ± 35.08	44.53	37.51	36.08	0.38
CheW	313	61.08 ± 30.90	44.56 ± 15.75	69.54 ± 25.52	50.59	35.35	36.70	0.40

^a^ Five weight-related traits: BW, body weight; TruW, trunk weight; WEC, weight excluding chelae; AppW, appendage weight; and CheW, cheliped weight. ^b^ Number of animals used for GWAS, ^c^ mean (±standard deviation), ^d^ coefficient of variation, ^e^ heritability value.

**Table 2:** Shared candidate SNPs and genes across all five traits.

Chr	Nsnp	QTL Region	Ngene
2	1	37,665,876–37,865,876	4
3	3	40,271,752–40,471,757	9
15	1	22,304,281–22,504,281	15
21	0	14,509,234–14,885,809	1
25	1	17,254,201–17,454,201	1
26	1	8,689,084–8,889,084	6
30	1	14,347,661–14,547,661	9
34	0	15,078,189–15,522,120	1
36	1	2,632,612–2,832,612	3

Chr, chromosome; Nsnp, number of shared candidate SNPs; QTL, quantitative trait locus; Ngene, number of shared candidate genes.

## Data Availability

The data presented in this study are available upon reasonable request from the corresponding author as they are currently being used in ongoing research projects.

## Supplementary Materials

The following supporting information can be downloaded at: https://www.mdpi.com/article/10.3390/ani15131829/s1, Figure S1: Correlation among the five traits of female and male; Figure S2: Sex difference analyses for five traits; Figure S3: Quantile–quantile (QQ) plots of GWAS results; Figure S4: Required sample sizes for linear multiple regression (fixed model) testing a single regression coefficient; Figure S5: LD block of candidate SNPs on chromosome 3; Table S1: Information on whole genome resequencing data; Table S2: Significant SNPs from GWAS for five traits; Table S3: Candidate genes identified by GWAS for five traits; Table S4: LD analysis of nine shared candidate SNPs; Table S5: GO analysis for candidate genes; Table S6: KEGG pathway analysis for candidate genes.
