# Supplementary material for: Genome-Wide Association Study for Weight-Related Traits in Scylla paramamosain Using Whole-Genome Resequencing
Source: Animals (Basel). 2025 Jun 20;15(13):1829. doi: 10.3390/ani15131829 (PMC12248745; doi:10.3390/ani15131829)
Supplement: Supplementary file 1 [file animals-15-01829-s001.zip › Supplementary Figures.pdf]

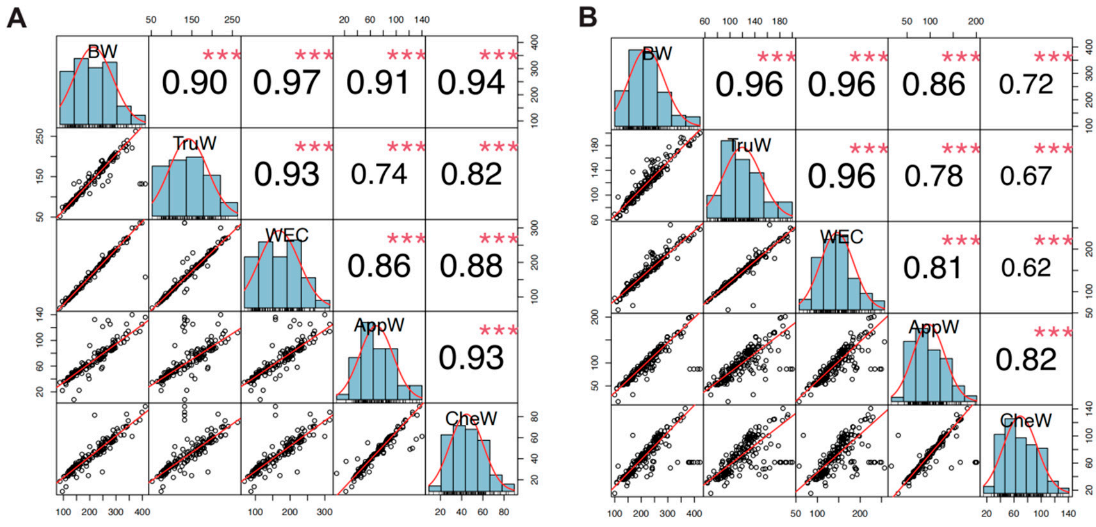

Figure S1 Correlation among the five traits of female and male. (A) Female; (B) Male. BW, body weight; TruW, trunk weight; WEC, weight excluding chelae; CW, cheliped weight; AppW, appendage weight.

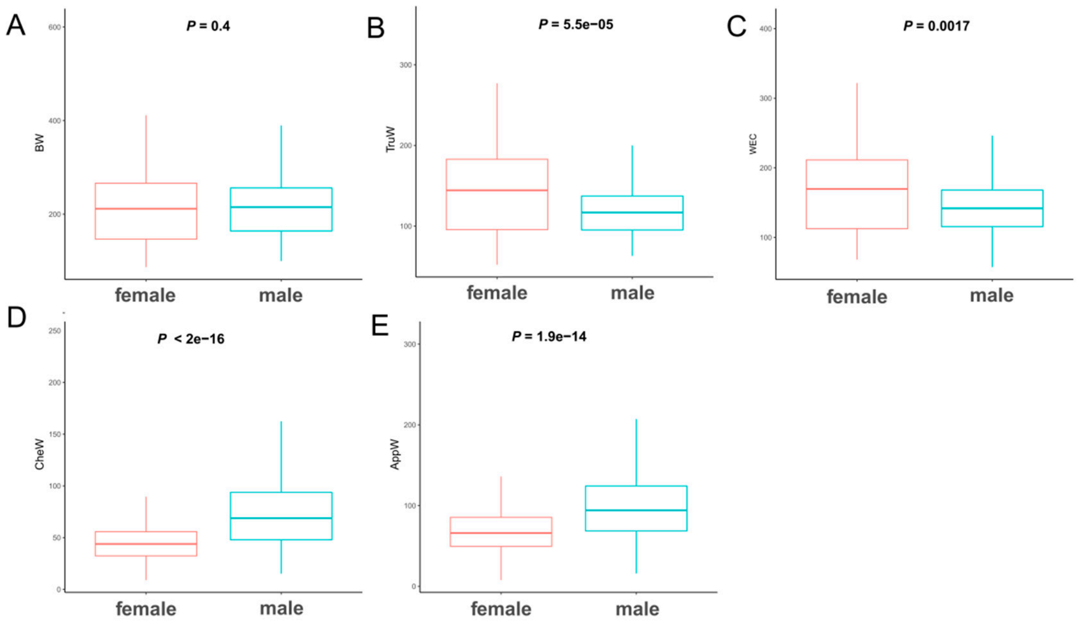

Figure S2 Sex difference analyses for five phenotypes. (A) Body weight (BW); (B) Trunk weight (TruW); (C) Weight excluding chelae (WEC); (D) Cheliped weight (CheW); (E) Appendage weight (AppW).

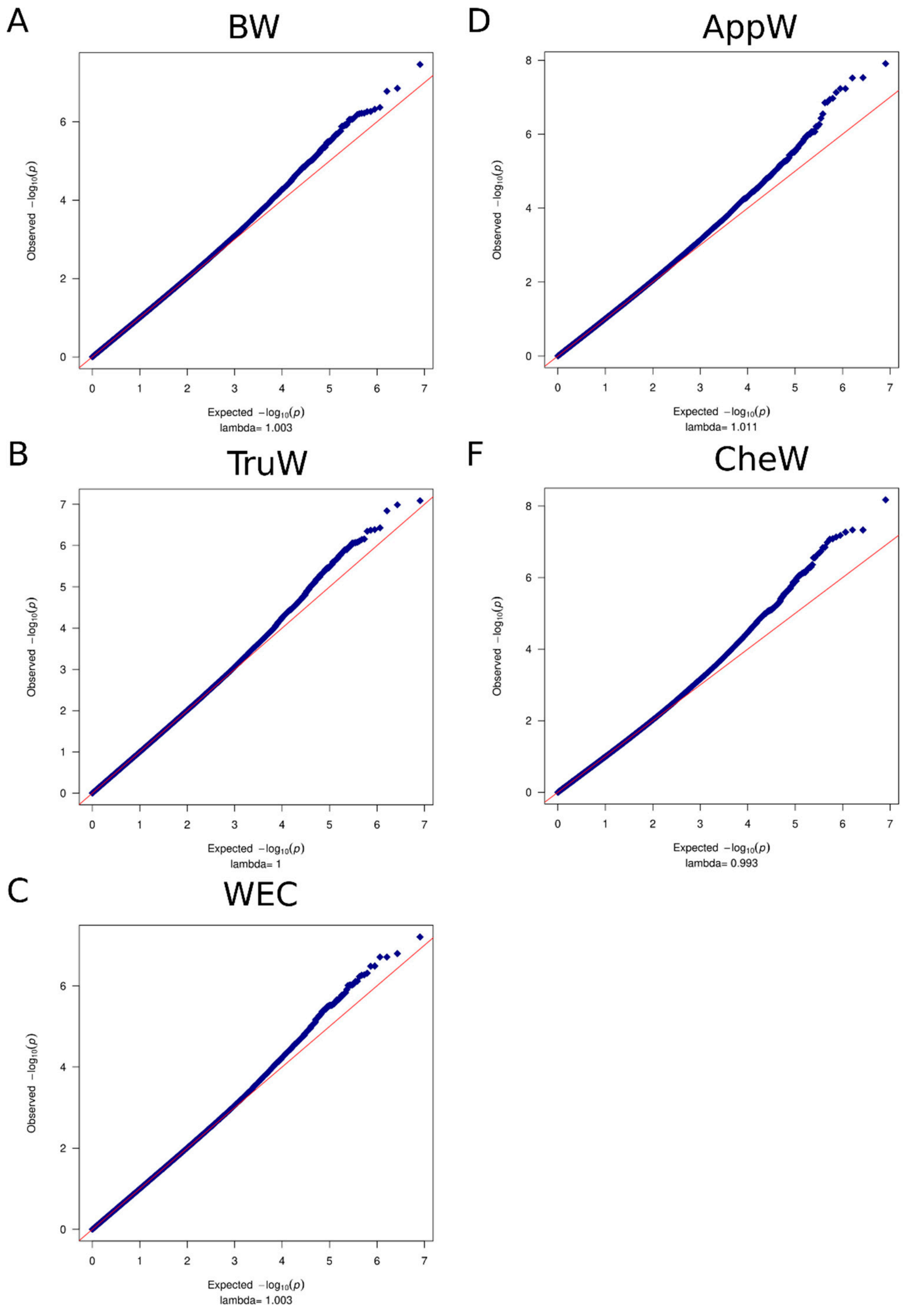

Figure S3 Quantile-Quantile (QQ) plot of GWAS results. (A) Body weight (BW); (B) Trunk weight (TruW); (C) Weight excluding chelae (WEC); (D) Appendage weight (AppW); (E) Cheliped weight (CheW).

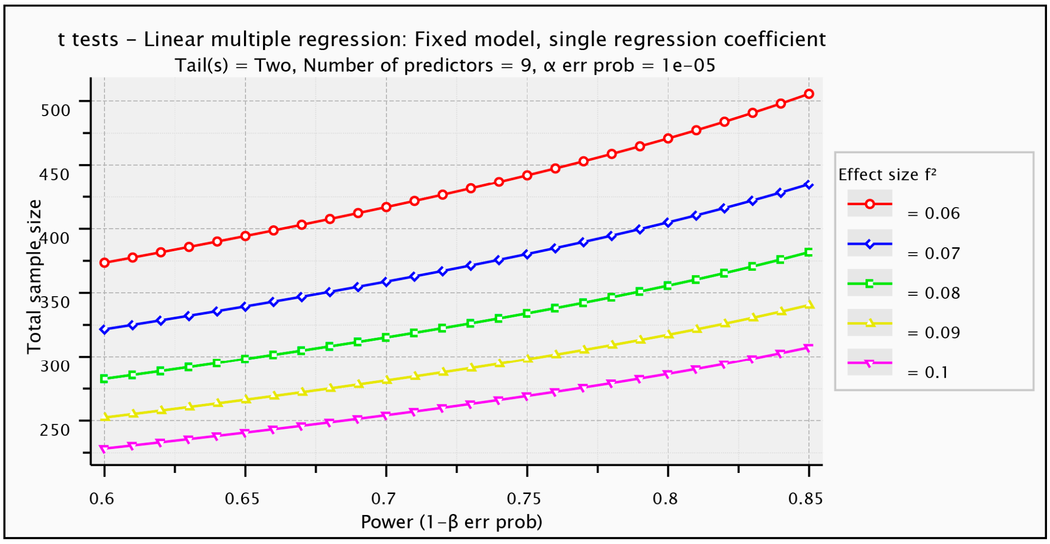

Figure S4 Required sample sizes for linear multiple regression (fixed model) testing a single regression coefficient. Analysis assumes a two-tailed test with 9 predictors and $\alpha = 10^{-5}$. Curves display statistical power (1-β) as a function of total sample size for effect sizes f^2 = 0.06–0.10. Power values range from 0.60 to 0.85 in 0.05 increments. Generated using G*Power software.

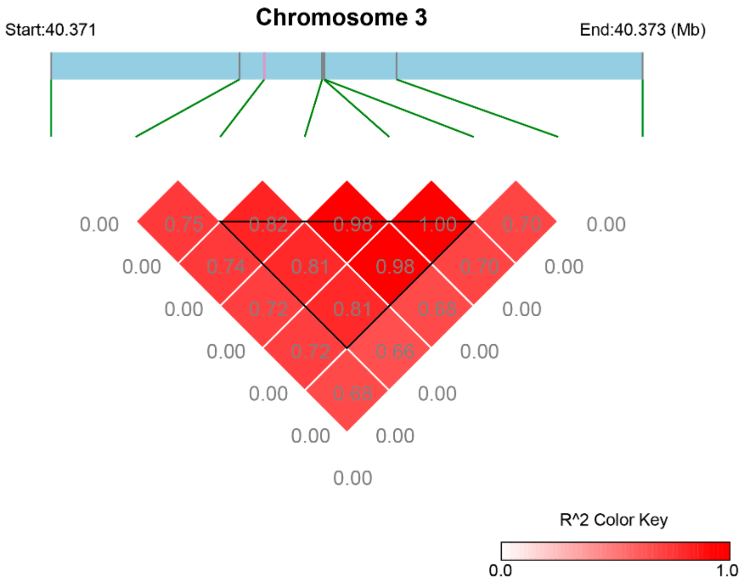

Figure S5 LD block of candidate SNPs on chromosome 3. Three SNPs shared by five weight-related traits located in a 143 bp (chr3: 40371614-40371757) LD block. Generated by LDBlockShow software.
